# Investigation of amyloid‐beta 42 levels in human nasal discharge fluid from healthy and Alzheimer’s discovers diagnostic values

**DOI:** 10.1002/alz.094935

**Published:** 2025-01-09

**Authors:** Da Hae Jung, Gowoon Son, Shengmin Wang, Hyun Kook Lim, Cheil Moon

**Affiliations:** ^1^ Convergence Research Advanced Centre for Olfaction, Daegu Korea, Republic of (South); ^2^ Daegu Gyeongbuk Institute of Science and Technology, Daegu Korea, Republic of (South); ^3^ University of Maastricht, Maastricht Netherlands; ^4^ Memory and Aging Center, UCSF Weill Institute for Neurosciences, University of California San Francisco, San Francisco, CA USA; ^5^ College of Medicine, The Catholic University of Korea, Seoul Korea, Republic of (South); ^6^ Yeouido St. Mary’s Hospital, College of Medicine, The Catholic University of Korea, Seoul Korea, Republic of (South)

## Abstract

**Background:**

Alzheimer’s disease (AD) therapy requires timely and accurate diagnosis for prompt drug intervention, emphasizing the need for a more accessible biomarker screening test. Previous studies identified higher expression of oligomeric Aβ in AD patients, however, the quantitative measurements of nasal Aβ42 levels of the full AD continuum remain unknown.

**Method:**

We collected nasal discharge samples from 161 subjects who underwent neuropsychological battery tests and amyloid‐PET and nasal Aβ42 levels were measured via enzyme‐linked immunosorbent assay (ELISA).

**Result:**

Our results revealed that the second‐highest quartile (Q3) group of nasal Aβ42 had the majority of patients with AD diagnosis, and those in the Q3 group exhibited the most impaired cognitive function with a strong significance. Nasal Aβ42 levels were also associated with brain amyloid PET results and increased risk of developing AD.

**Conclusion:**

Our findings suggest that nasal Aβ42 is a promising biomarker for AD diagnosis and risk assessment.

